# Autophagy-enhancing drugs limit mucosal HIV-1 acquisition and suppress viral replication ex vivo

**DOI:** 10.1038/s41598-021-84081-4

**Published:** 2021-02-26

**Authors:** Alexandra P. M. Cloherty, Nienke H. van Teijlingen, Tracy-Jane T. H. D. Eisden, John L. van Hamme, Anusca G. Rader, Teunis B. H. Geijtenbeek, Renée R. C. E. Schreurs, Carla M. S. Ribeiro

**Affiliations:** 1 Amsterdam UMC, University of Amsterdam, Department of Experimental Immunology, Amsterdam institute for Infection & Immunity, Meibergdreef 9, Amsterdam, The Netherlands; 2 Amsterdam UMC, Vrije Universiteit Amsterdam, Department of Medical Oncology, Cancer Center Amsterdam, De Boelelaan 1117, Amsterdam, The Netherlands

**Keywords:** HIV infections, Autophagy, Antivirals, Experimental models of disease

## Abstract

Current direct-acting antiviral therapies are highly effective in suppressing HIV-1 replication. However, mucosal inflammation undermines prophylactic treatment efficacy, and HIV-1 persists in long-lived tissue-derived dendritic cells (DCs) and CD4^+^ T cells of treated patients. Host-directed strategies are an emerging therapeutic approach to improve therapy outcomes in infectious diseases. Autophagy functions as an innate antiviral mechanism by degrading viruses in specialized vesicles. Here, we investigated the impact of pharmaceutically enhancing autophagy on HIV-1 acquisition and viral replication. To this end, we developed a human tissue infection model permitting concurrent analysis of HIV-1 cellular targets ex vivo. Prophylactic treatment with autophagy-enhancing drugs carbamazepine and everolimus promoted HIV-1 restriction in skin-derived CD11c^+^ DCs and CD4^+^ T cells. Everolimus also decreased HIV-1 susceptibility to lab-adapted and transmitted/founder HIV-1 strains, and in vaginal Langerhans cells. Notably, we observed cell-specific effects of therapeutic treatment. Therapeutic rapamycin treatment suppressed HIV-1 replication in tissue-derived CD11c^+^ DCs, while all selected drugs limited viral replication in CD4^+^ T cells. Strikingly, both prophylactic and therapeutic treatment with everolimus or rapamycin reduced intestinal HIV-1 productive infection. Our findings highlight host autophagy pathways as an emerging target for HIV-1 therapies, and underscore the relevancy of repurposing clinically-approved autophagy drugs to suppress mucosal HIV-1 replication.

## Introduction

Globally, there are 1.7 million new HIV infections per year, and 37.9 million people living with HIV (PLWH) as of 2018^1^. HIV-1 therefore continues to be a major global health concern despite the advent of effective prophylactic and therapeutic antiretroviral therapies. HIV-1 transmission typically occurs at vaginal or gastrointestinal mucosa^2,3^. At stratified squamous epithelia such as skin and mucosa, HIV-1 virions encounter CD4^+^ T cells, dendritic cell (DC) subsets, and macrophages^4–7^. Seminal studies have shown that subepithelial CD4^+^ T cells and DCs expressing surface receptors DC-SIGN or Siglec-1 are key actors in the early establishment of HIV-1 entry and infection in human tissues^6,8,9^. Migratory CD11c^+^CD14^+^ DCs in human tissue are hijacked by HIV-1, to promote HIV-1 spread to lymphatic tissue where the virus is efficiently transmitted from DCs to uninfected CD4^+^ T cells^4–6,9^. Likewise, tissue CD4^+^ T cells have been demonstrated to migrate out of mucosal epithelia, thereby contributing to amplification of local infection and systemic HIV-1 dissemination^8^.

Although HIV-1 pre-exposure prophylaxis (PrEP) therapy is highly efficacious in preventing HIV-1 infection of mucosal DC subsets and T cells, recent studies have demonstrated that inflammation of mucosal tissues undermines the effectiveness of PrEP^10,11^. Maturation and migration of DC subsets increases their susceptibility to HIV-1 infection, which may contribute to the reduced PrEP efficacy during mucosal inflammation^12–14^. Novel prophylactic strategies to enhance anti-HIV-1 immunity are therefore required to address prevention needs of diverse and high-risk populations.

Therapeutic antiretroviral therapies are highly effective and have transformed the lives of HIV-1 patients by decreasing viral burden, prolonging life expectancy, and greatly increasing quality of life of PLWH. However, contemporary combination antiretroviral therapy (cART) is not curative and treated HIV-1 patients still suffer from severe comorbidities due to persistent inflammation, and residual viral replication in cellular reservoirs of HIV-1^15–17^. Latent HIV-1 persists in long-lived tissue-derived myeloid cells and CD4^+^ T cells, and reactivates upon cessation of cART, leading to transfer of HIV-1 to uninfected neighbouring cells^17–19^. In particular, intestinal mucosa and gut associated lymphoid tissue (GALT) represent major reservoirs in which HIV-1 latently persists and replicates^16,17^. Furthermore, as with other direct-acting antiviral therapies, cART regimens are associated with emergence of drug resistant HIV-1 variants^20^. Eradication of HIV-1 reservoirs in long-lived cells thus remains one of the greatest challenges in HIV-1 care research, and there is an urgent need to identify new therapeutic strategies to curtail residual HIV-1 replication.

Host-directed therapy is an emerging approach for treating infectious diseases^20^. Here, we have focused on boosting innate host immune defense mechanisms, with a focus on the autophagy pathway, in order to suppress HIV-1 acquisition and residual virus replication.

Autophagy is an intracellular degradation process that also functions as an innate defense mechanism, in which specialized double-membrane vesicles form to envelop cytosolic cargo such as viruses and target them for lysosomal degradation. Langerhans cells (LCs), a subset of human DCs inhabiting epithelial tissues, are intrinsically resistant to HIV-1. We were the first to establish that an autophagy mechanism confers cell-mediated resistance to HIV-1^13,14,21,22^. In LCs, human TRIM5α forms a complex with an Atg16L1-Atg5 autophagy scaffold after viral fusion, which targets the HIV-1 capsid for lysosomal degradation via autophagy, thereby preventing infection of LCs. Contrastingly, other subepithelial DCs and CD4^+^ T cells lack this intrinsic autophagy-based restriction mechanism. We and others have shown that HIV-1 escapes autophagy-mediated restriction in CD11c^+^ DCs and CD4^+^ T cells^22–26^. Importantly, enhancement of autophagy has also been associated with better prognosis of HIV-1 infected individuals^27,28^. In long-term nonprogressor PLWH, ex vivo peripheral blood mononuclear cells (PBMCs) have been shown to exhibit more specialized autophagy vesicles than those from normal progressors^27^. Furthermore, in cART-treated HIV-1 patients and animal models, autophagy dysfunction was associated with increased risk for HIV-1 associated comorbidities such as cardiomyopathies and neurodegenerative diseases^28,29^. Collectively, these findings underline the protective role for autophagy in HIV-1 acquisition and HIV-1 disease progression.

Autophagy is pharmacologically employed to treat diverse non-infectious conditions, including treatment of epilepsy, cancers, and transplantation^30–32^. Here, we investigated whether pharmacological enhancement of the autophagy pathway limits HIV-1 replication in diverse HIV-1 cellular targets. To this end, we developed a tissue infection model that enabled us to identify clinically approved autophagy drugs that suppress both HIV-1 acquisition as well as HIV-1 replication in human tissue-derived cells implicated in HIV-1 spread throughout the body. We show that HIV-1 replication in LCs, CD11c^+^ DCs, and CD4^+^ T cells can be suppressed by either prophylactic or therapeutic exposure to selected autophagy-enhancing drugs. Furthermore, selected autophagy-enhancing drugs also decreased HIV-1 susceptibility and productive HIV-1 infection in relevant mucosal tissues, namely vaginal and intestinal mucosal tissues. Our findings underscore the therapeutic potential of repurposing clinically approved autophagy drugs to increase cell-mediated resistance to HIV-1 and suppress residual HIV-1 replication and extracellular virus release by primary human tissue-derived cells.

## Results

### Autophagy-enhancing drugs decrease HIV-1 susceptibility of primary activated human LCs

Recently, we have outlined the autophagy-dependent mechanism directing HIV-1 restriction by primary immature LCs^21^. However, during inflammation of tissues, activated LCs migrate, and become susceptible to HIV-1, thereby promoting viral dissemination and transmission to CD4^+^ T cells^12–14,33,34^.

**Figure 1:**
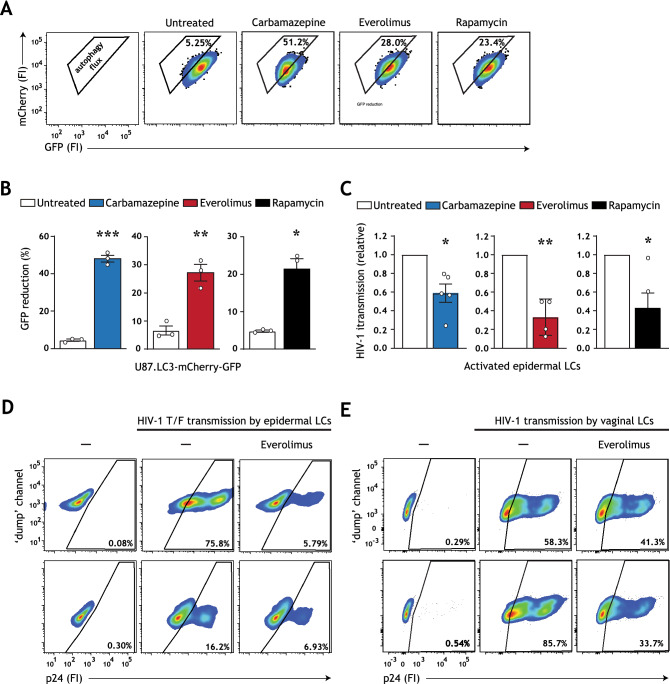
Carbamazepine, everolimus, and rapamycin induce autophagy flux and decrease HIV-1 susceptibility. (**A**,**B**) U87 cell line expressing tandem fluorescent-tagged LC3 (mCherry-GFP-LC3) was incubated with carbamazepine (1000 μM), everolimus (30 nM), or rapamycin (100 nM) for 48 h. The GFP tag is acid-sensitive and is quenched upon autophagosome acidification. Autophagy flux was determined by the % GFP signal reduction. Representative flow cytometry plots (**A**) and quantification (**B**), data are mean ± SE of n = 3 experiments measured in duplicate. Open circles represent the mean of duplicates from each independent experiment. *P < 0.05, **P < 0.01, ***P < 0.001, Student's *t* test. (**C**) Human epidermal biopsies were prophylactically treated with carbamazepine (100 μM), everolimus (30 nM), or rapamycin (100 nM), or left untreated, and subsequently infected with HIV-1 NL4.3 BaL for 72 h. Emigrated LCs were washed, and co-cultured with U87.CD4.CCR5 cells. HIV-1 transmission by LCs was assessed in LC-U87.CD4.CCR5 co-culture for 72 h, determined by intracellular p24 staining by flow cytometer. LC-marker CD1a was used to exclude single LCs and LC-U87 conjugates from analysis. A detailed graphical depiction of HIV-1 transmission across epidermal tissue is available in Figure S2A, Supplementary Information. Data are mean ± SE of n = 5 experiments measured in duplicate. Open circles represent the mean of duplicates from each independent experiment. *P < 0.05, **P < 0.01, one-sample *t* test. (**D**) Human epidermal biopsies were pre-treated with everolimus (30 nM), followed by infection with transmitted/founder HIV-1 THRO for 72 h. Emigrated LCs were washed, and co-cultured with U87.CD4.CCR5 cells. HIV-1 transmission by LCs was assessed in LC-U87.CD4.CCR5 co-culture for 72 h, determined by intracellular p24 staining by flow cytometer. LC-marker CD1a was used to exclude single LCs and LC-U87 conjugates from analysis. Flow cytometry plots of HIV-1 infection of U87.CD4.CCR5 cells for each epidermal tissue donor is shown. (**E**) Isolated human immature vaginal LCs, obtained by CD1a magnetic cell isolation, were pre-treated with everolimus (30 nM) and subsequently infected with HIV-1 NL4-3 BaL for 72 h. Vaginal LCs were washed, and co-cultured with U87.CD4.CCR5 cells. HIV-1 transmission by LCs was assessed in LC-U87.CD4.CCR5 co-culture for 72 h, determined by intracellular p24 staining by flow cytometer. LC-marker CD1a was used to exclude single LCs and LC-U87 conjugates from analysis. Flow cytometry plots of HIV-1 infection of U87.CD4.CCR5 cells for each vaginal LC donor is shown.

Here, we aimed to evaluate whether autophagy-enhancing strategies can limit HIV-1 transmission by activated LCs. To this end, we first screened for the potential of FDA-approved drugs to enhance autophagy flux, i.e. autophagy-mediated degradation via the lysosome, in an engineered U87 human cell line. In the standardized U87.LC3-mCherry-GFP autophagy reporter system, reduction in GFP fluorescence is representative of enhanced autophagy flux^35^. All analyzed autophagy drugs strongly enhanced autophagy flux (Fig. 1A,B). Next, epidermal tissue biopsies were pre-incubated with optimized concentrations of carbamazepine, everolimus and rapamycin (Fig. S1A–E, Supplementary Information), and subsequently infected with HIV-1. In concordance with previous reports^13,21,33^ activated emigrated LCs were more susceptible to HIV-1 infection, and thereby transmitted HIV-1 to target U87.CD4.CCR5 cells (Figure S2A,B, Supplementary Information). Notably, HIV-1 transmission by emigrated LCs was reduced by treatment with each of the drugs **(**Fig. 1C). These data underline that a broad spectrum of autophagy-enhancing drugs have the potential to revamp resistance to HIV-1 infection in activated LCs.

We have recently shown that sexually transmitted/founder (T/F) HIV-1 viruses are relatively resistant to Langerhans cell-mediated restriction^12^. Notably, pre-treatment with everolimus prevented transmission of HIV-1 T/F virus by emigrated primary human LCs (Fig. 1D). Finally, in line with previous reports, vaginal LCs resembled epidermal counterparts^12,13^ and everolimus pretreatment also prevented HIV-1 transmission by vaginal LCs (Fig. 1E). Collectively, these data further underscore the potential of autophagy mechansisms in curbing HIV-1 acquisition by human primary LCs.

### *Prophylactic treatment with autophagy drugs reduces HIV-1 infection of tissue-derived DC subsets and CD4*^+^*T cells*

Subepithelial tissue-derived CD11c^+^ DCs, in particular CD11c^+^CD14^+^ DCs, are susceptible to HIV-1 and hijacked by the virus to migrate to lymphatic tissues where they efficiently transmit HIV-1 to CD4^+^ T cells^4–6,9^. We and others have shown that HIV-1 escapes autophagy in both CD11c^+^ DCs and T cells^22–24^. Here, we investigated whether pharmacological enhancement of autophagy has the potential to decrease susceptibility of these cells to HIV-1.

**Figure 2:**
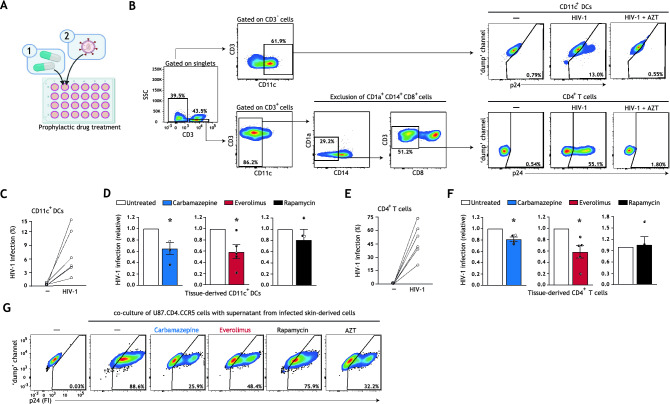
Prophylactic treatment with autophagy drugs reduces HIV-1 acquisition by tissue-derived DCs and CD4^+^ T cells. (**A**) Brief schematic representation of the novel HIV-1 tissue infection model for drug screening; an extended graphical representation of the prophylactic treatment model is available in Figure S3A, Supplementary Information. Biopsies including epithelium and subepithelium were taken from skin and cultured in a 24 well plate. Skin biopsies were prophylactically treated with autophagy drugs and subsequently infected with HIV-1. HIV-1 infection of different cell types and subsets was quantified using multiparameter flow cytometry. (**B**) Gating strategy utilized to discriminate HIV-1 infection in the emigrated CD11c^+^ DCs and CD4^+^ T cells. Treatment of tissue biopsies with HIV-1 replication inhibitor AZT (zidovudine, 20 μM) confirmed productive HIV-1 infection of these cell types. A detailed gating strategy can be found in Figure S4, Supplementary Information. (**C**–**F**) Human tissue biopsies were prophylactically treated with carbamazepine (100 μM), everolimus (5 nM), or rapamycin (100 nM) for 15 h, or left untreated, and subsequently infected with HIV-1 NL4.3BaL for 48 h. Emigrated cells were then harvested, washed, and replated for an additional 72 h. HIV-1 infection of emigrated CD11c^+^DCs was determined as the percentage of CD3^−^CD11c^+^p24^+^ cells (**C**,**D**), and HIV-1 infection of emigrated CD4^+^ T cells as the percentage of CD3^+^CD11c^−^CD14^−^CD1a^−^CD8^−^p24^+^ cells (**E**,**F**), determined by flow cytometer. (**C**–**F**) Data are mean ± SE of n = 4–5 donors measured in duplicate. Open circles represent the mean of duplicates from each independent experiment. (**D**,**F**) Untreated HIV-1 infected cells was set at 1 *P < 0.05; one-sample *t* test. (**G**) Skin biopsies including epithelium and subepithelium were prophylactically treated for 15 h with autophagy drugs carbamazepine (100 μM), everolimus (5 nM), rapamycin (100 nM), HIV-1 replication inhibitor AZT (zidovudine, 20 μM), or left untreated, followed by infection with HIV-1 NL4.3BaL. 36 h post-infection, emigrated tissue-derived cells were extensively washed to remove input virus, and replated in new medium in a 96-well plate. Supernatant from infected tissue-derived cells was collected 120 h after replating and co-cultured with U87.CD4.CCR5 cells for 72 h, to further confirm productive HIV-1 infection. A detailed graphical representation of this extracellular virus release assay is available in Figure S3B, Supplementary Information. HIV-1 infection of U87.CD4.CCR5 cells was determined by intracellular p24 staining by flow cytometer. Representative (n = 2 tissue donors) flow cytometry plots of HIV-1 infection of U87.CD4.CCR5 cells incubated with supernatants from HIV-1 infected skin-derived cells is shown.

To this end, we developed a novel tissue HIV-1 infection model that allows for multi-drug screenings ex vivo (schematic representation in Fig. S3A, Supplementary Information). Our tissue model includes major target cells of HIV-1 that are able to migrate out of tissues, and are implicated in HIV-1 dissemination. Human skin biopsies including epithelium and subepithelium were prophylactically treated with optimized concentrations of carbamazepine, everolimus, or rapamycin, and subsequently infected with HIV-1 (Figs. 2A, S1A–E, Supplementary Information). Infection of different cell types and subsets was quantified using multiparameter flow cytometry (Fig. 2B, detailed gating strategy in Fig. S4, Supplementary Information). Productive HIV-1 infection of human skin biopsies was confirmed by AZT treatment (Figs. 2B, S4, Supplementary Information). Autophagy enhancement by drug treatment was confirmed by assessing intracellular LC3-II accumulation in skin-derived CD11c^+^ DCs and CD4^+^ T cells via multiparameter flow cytometry^36,37^ (Fig. S5A,B, Supplementary Information).

In concordance with previous reports^6,38^, CD11c^+^ DCs and CD4^+^ T cells were productively infected with HIV-1 at different rates (Fig. 2C,E) and CD11c^+^CD14^+^CD1a^−^ DCs were the primary subepithelial DC subset productively infected by HIV-1 (Fig. S4, Supplementary Information**)**. Notably, prophylactic treatment with carbamazepine or everolimus decreased HIV-1 infection of both CD11c^+^ DCs and CD4^+^ T cells (Fig. 2D,F). Although prophylactic treatment with rapamycin decreased HIV-1 susceptibility of epithelial LCs (Fig. 1C), it did not reduce infection of subepithelial CD11c^+^ DCs or CD4^+^ T cells (Fig. 2D,F). Next, we assessed extracellular virus production by incubating permissive U87.CD4.CCR5 cells with the supernatant of prophylactically treated emigrated tissue-derived cells that had been extensively washed to remove input virus (schematic representation in Fig. S3B, Supplementary Information). Prophylactic treatment with carbamazepine or everolimus also reduced production of extracellular virus (Fig. 2G). These data indicate that pharmaceutically enhancing autophagy is a feasible strategy for limiting HIV-1 acquisition and extracellular virus release by otherwise HIV-1 permissive cells.

### Therapeutic treatment with autophagy drugs limits ongoing HIV-1 replication in a cell-specific manner

Although cART is highly effective in decreasing viral replication, treated HIV-1 patients exhibit residual viral replication in long-lived tissue-resident cell types like DCs and CD4^+^ T cells^16–18^. There is therefore a dire need to identify new intervention strategies to limit tissue-derived HIV-1 reservoirs. Here, we assessed whether enhancing autophagy flux could limit viral replication in ongoing HIV-1 infection in ex vivo human tissues.

**Figure 3:**
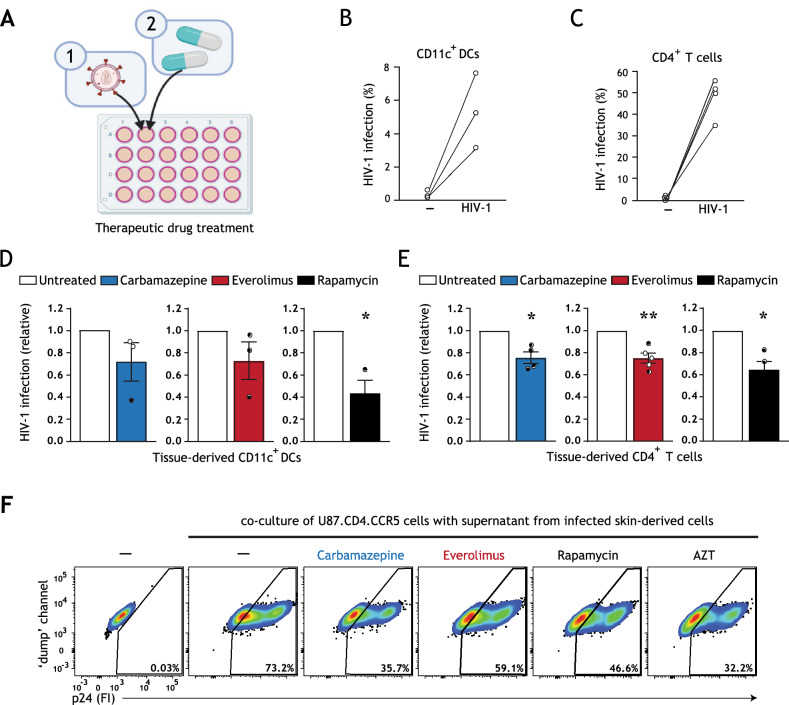
Therapeutic treatment with autophagy drugs reduces HIV-1 replication in a cell-specific manner. (**A**) Brief schematic representation of HIV-1 tissue infection model for drug screening, an extended graphical representation of the therapeutic treatment model is available in Figure S3C, Supplementary Information. Biopsies including epithelium and subepithelium were taken from human skin tissue, infected with HIV-1 NL4.3BaL for 15 h, and subsequently treated with autophagy drugs. HIV-1 infection of different cell types and subsets was quantified using multiparameter flow cytometry. A detailed gating strategy can be found in Supplementary Information Figure S4. (**B**–**E**) Human tissue biopsies were therapeutically treated with carbamazepine (100 μM), everolimus (5 nM), or rapamycin (100 nM), or left untreated. HIV-1 infection of emigrated CD11c^+^DCs was determined as the percentage of CD3^−^CD11c^+^p24^+^ cells (**B**,**D**), and HIV-1 infection of emigrated CD4^+^ T cells as the percentage of CD3^+^CD11c^−^CD14^−^CD1a^−^CD8^−^p24^+^ cells (**C**,**E**), determined by flow cytometer. (**B**–**E**) Data are mean ± SE of n = 3–5 donors measured in duplicate. Open circles represent the mean of duplicates from each independent experiment. (**D**,**E**) Untreated, HIV-1 infected cells was set at 1, *P < 0.05, **P < 0.01, one-sample *t* test. (**F**) Skin biopsies including epithelium and subepithelium were infected with HIV-1 NL4.3BaL for 15 h. Subsequently, skin biopsies were therapeutically treated with autophagy drugs carbamazepine (100 μM), everolimus (5 nM), rapamycin (100 nM), HIV-1 replication inhibitor AZT (zidovudine, 20 μM), or left untreated. 36 h after treatment, emigrated tissue-derived cells were extensively washed to remove input virus, and replated in new medium in a 96-well plate. Supernatant from infected tissue-derived cells was collected 120 h following replating and co-cultured with U87.CD4.CCR5 cells for 72 h, to further confirm productive HIV-1 infection. A detailed graphical representation of this extracellular virus release assay is available in Figure S3D, Supplementary Information. HIV-1 infection of U87.CD4.CCR5 cells was determined by intracellular p24 staining by flow cytometer. Representative (n = 2 tissue donors) flow cytometry plots of HIV-1 infection of U87.CD4.CCR5 cells incubated with supernatants from HIV-1 infected skin-derived cells is shown.

In these experiments, we utilized our tissue HIV-1 infection model (schematic representation in Fig. S3C, Supplementary Information). Skin biopsies were first infected with HIV-1, and subsequently treated with optimized concentrations of the selected autophagy drugs (Figs. 3A, S1A–E, S5A,B, Supplementary Information). Infection of different cell types was quantified using multiparameter flow cytometry as previously detailed (Fig. 3B,C), and CD11c^+^ DCs and CD4^+^ T cells were productively infected as evidenced by an infection block under AZT treatment (Fig. S4, Supplementary Information).

Notably, we observed a cell-specific effect of therapeutic treatment with autophagy drugs. Therapeutic treatment with rapamycin, but not carbamazepine nor everolimus, significantly reduced HIV-1 infection in tissue-derived CD11c^+^ DC subsets (Fig. 3D). Therapeutic everolimus treatment was also efficient in blood-derived DC subsets (Fig. S6, Supplementary Information). In contrast, HIV-1 infection of CD4^+^ T cells was diminished by therapeutic treatment with all selected drugs (carbamazepine, rapamycin *P* < 0.05; everolimus *P* < 0.01) (Fig. 3E). Furthermore, we confirmed the overall inhibitory effect of therapeutic treatment with the selected autophagy drugs on extracellular HIV-1 release by skin-derived cells (Fig. 3F, schematic representation in Fig. S3D, Supplementary Information). Altogether, our data underline the importance of concurrent assessment of potential antiviral therapies on diverse HIV-1-susceptible cell types and demonstrate that enhancing autophagy flux with selected drugs is an efficacious strategy for suppressing ongoing HIV-1 replication and virus release in tissue-derived cells.

### Treatment with everolimus or rapamycin reduces HIV-1 replication in intestinal CD4^+^ T cells

**Figure 4:**
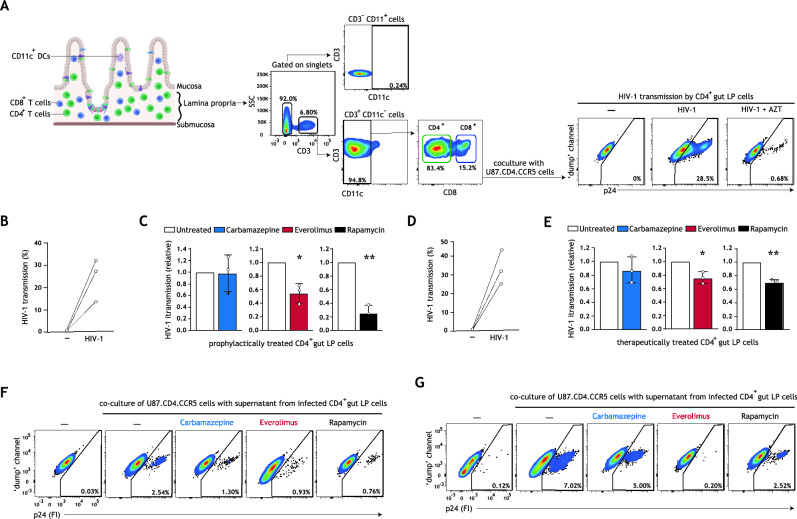
Autophagy-enhancing drugs everolimus and rapamycin limit intestinal HIV-1 transmission and reduce ongoing HIV-1 replication in intestinal CD4 ^+^ T cells. (**A**) Human intestinal lamina propria lymphocytes (LPLs) were isolated as previously described^40^. Gating strategy employed to identify gut CD4 ^+^ T cells is shown; an extended gating strategy is available in Figure S7D, Supplementary Information. Following HIV-1 infection, LPLs were washed extensively to remove input virus, and subsequently co-cultured with permissive U87.CD4.CCR5 cells for 72 h. HIV-1 transmission by gut LPLs was determined by intracellular p24 staining; treatment of LPLs with HIV-1 replication inhibitor AZT (zidovudine, 20 μM) confirmed productive intestinal HIV-1 infection. (**B**,**C**) LPLs were prophylactically treated with carbamazepine (100 μM), everolimus (5 nM), or rapamycin (100 nM) for 15 h, or left untreated, followed by infection with HIV-1 NL4.3BaL for 24 h. Gut LPLs were harvested and washed to remove input virus, and subsequently co-cultured with U87.CD4.CCR5 cells. HIV-1 transmission by LPLs was assessed in LPL-U87.CD4.CCR5 co-culture for 72 h, determined by intracellular p24 staining by flow cytometer. T cell-marker CD3 was used to exclude single LPLs and LPL-U87.CD4.CCR5 conjugates from analysis. A detailed graphical depiction of HIV-1 transmission by gut LPLs is available in Supplementary Information Figure S7E. Data are mean ± SE of n = 3 donors measured in duplicate. Open circles represent the mean of duplicates from each independent experiment. Untreated, HIV-1 infected cells was set at 1, *P < 0.05, **P < 0.01, one-sample *t* test. (**D**,**E**) LPLs were infected with HIV-1 NL4.3BaL for 15 h, and subsequently therapeutically treated with carbamazepine (100 μM), everolimus (5 nM), or rapamycin (100 nM) for 24 h, or left untreated. Gut LPLs were harvested and washed to remove input virus, and subsequently co-cultured with U87.CD4.CCR5 cells. HIV-1 transmission by LPLs was assessed in LPL-U87.CD4.CCR5 co-culture for 72 h, determined by intracellular p24 staining by flow cytometer. T cell-marker CD3 was used to exclude single LPLs and LPL-U87.CD4.CCR5 conjugates from analysis. A detailed graphical depiction of HIV-1 transmission by gut LPLs is available in Figure S7F, Supplementary Information. (**F**) Following prophylactic treatment and infection with HIV-1 NL4.3BaL (as in **B**,**C**), LPLs were extensively washed to remove input virus and replated in new medium in a 96-well plate. (**G**) Following infection with HIV-1 NL4.3BaL and therapeutic treatment (as in **D**,**E**), LPLs were extensively washed to remove input virus and replated in new medium in a 96-well plate. (**F**,**G**) Supernatants from infected intestinal prophylactically—(**F**) or therapeutically—(**G**) treated LPLs were collected 24 h after replating and co-cultured with U87.CD4.CCR5 cells for 72 h, to further confirm productive intestinal HIV-1 infection. HIV-1 infection of U87.CD4.CCR5 cells was determined by intracellular p24 staining by flow cytometer. Representative (n = 2 tissue donors) flow cytometry plots of HIV-1 infection of U87.CD4.CCR5 cells incubated with supernatants from HIV-1 infected gut LPLs.

Intestinal mucosa represents a major tissue reservoir in which HIV-1 latently persists, even in treated HIV-1 patients^16,17^. Additionally, intestinal mucosa is an important entry site for HIV-1 during mother-to-child or sexual transmission^2,3^. Importantly, memory CD4^+^ T cells are abundant in infant and fetal mucosa (Fig. S7A, Supplementary Information), thus providing a large pool of cells susceptible to HIV-1 infection and a long-lived HIV-1 cellular reservoir that reactivates upon cessation of cART^17,19,39,40^. Here, we investigated whether prophylactic or therapeutic treatment with carbamazepine, everolimus, or rapamycin could limit HIV-1 replication across mucosal tissue. As isolated human intestinal fetal lamina propria lymphocytes (LPLs) have a limited cell survival in long-term cultures (S7C, Supplementary Information), we have studied intestinal HIV-1 productive infection by carrying out HIV-1 transmission by LPLs in co-culture with target U87.CD4.CCR5 cells (Fig. 4A, schematic representation in Fig. S7E,F, Supplementary Information). Intestinal LPLs, composed primarily of CD4^+^ T cells, were treated prophylactically or therapeutically with optimized concentrations of carbamazepine, everolimus, or rapamycin (Figs. S5C, S7B,D, Supplementary Information). Strikingly, prophylatic treatment with everolimus or rapamycin significantly decreased HIV-1 acquisition by intestinal lymphocytes (Fig. 4B,C). Similarly, therapeutic treatment with everolimus or rapamycin significantly reduced HIV-1 transmission by intestinal LPLs across three donors (Fig. 4D,E). Finally, prophylactic and therapeutic treatment with everolimus or rapamycin additionally resulted in decreased extracellular HIV-1 release by intestinal LPLs (Fig. 4F,G). These data strongly suggest that pharmacological enhancement of autophagy, potentially in combination with existing cART regimens, may be a powerful strategy to prevent and intervene in ongoing HIV-1 replication in mucosal CD4^+^ T cells.

## Discussion

Our data highlights the prophylactic and therapeutic potential of boosting host autophagy to intervene in HIV-1 infections. Host-directed therapies are a promising emerging approach in addressing infectious diseases, and may have the potential to both interfere with host cell mechanisms hijacked by viruses for productive infection, and boost innate immune defense mechanisms^20,22^. A major advantage of host-directed therapies above direct acting antivirals is the reduced likelihood of emergence of drug-resistant variants^20^. We propose that repurposing autophagy drugs has the potential to improve outcomes of HIV-1 therapies.

Here, the development of a novel HIV-1 tissue infection model permitted screening of clinically approved autophagy drugs in both pre-exposure (Fig. 2A) and post-exposure (Fig. 3A) settings. This model is unique in that it permitted concurrent analysis of multiple HIV-1 cellular targets (Fig. S4, Supplementary Information), thus providing an efficient model for identification of broadly active host-directed therapies. We have shown that pre-treatment with autophagy-enhancing drug everolimus, an FDA-approved cancer chemotherapeutic, potently controlled replication of both lab-adapted and T/F HIV-1 strains (Fig. 1C–E). Prophylactically enhancing autophagy revamped HIV-1 restriction in emigrated activated LCs (Figs. 1C,D, S2B, Supplementary Information), and limited HIV-1 acquisition by otherwise permissive CD11c^+^ DC subsets (Fig. 2C,D) and CD4^+^ T cells (Fig. 2E,F). In addition, prophylactic treatment of tissue-derived cells with carbamazipine or everolimus decreased the overall production of extracellular virus (Fig. 2G). Altogether, these results indicate that enhancing autophagy flux represents a pertinent strategy for preventing HIV-1 transmission by multiple tissue-derived target cells.

Notably, we observed that treatment with specific autophagy drugs is associated with cell-specific effects. While therapeutic treatment with rapamycin, clinically utilized as an immunosuppressant for transplantation patients, significantly reduced HIV-1 infection in tissue-derived CD11c^+^ DC subsets, therapeutic treatment with everolimus and carbamazepine did not (Fig. 3D). This is noteworthy as everolimus is an analogue of rapamycin. It is acknowledged that the primary target of rapamycin is mTOR complex-1 (mTORC1), whereas everolimus is a more potent inhibitor of mTORC2 and additionally inhibits ERK phosphorylation upstream of mTOR functioning in the autophagy pathway^32,41^. The distinct mechanisms of action of rapamycin and everolimus could contribute to the different effects of these drugs on different cell types. Similarly, while prophylactic treatment with carbamazepine, FDA-approved as an anti-epileptic drug, limited HIV-1 acquisition by tissue-derived CD11c^+^ DCs and CD4^+^ T cells (Fig. 2D,F), therapeutic treatment with carbamazepine reduced HIV-1 replication in CD4^+^ T cells but not CD11c^+^ DCs (Fig. 3D,E). As all autophagy drugs utilized in this model indeed induced autophagy flux (Figs. 1A,B, S5A,B, Supplementary Information) and did not impact virus entry (Fig. S8, Supplementary Information), these data may reflect cell-specific differences in drug uptake or tissue penetrance, which has also been suggested for other direct-acting antiviral therapies^42,43^. Notably, everolimus has been demonstrated to have both higher systemic bioavailability (in rats) and a shorter time to peak tissue concentration (in humans) as compared to rapamycin^32^. Everolimus was also recently identified to be more efficacious than rapamycin in ameliorating inflammation in vivo in mice, which was potentially mediated by increased inhibition of ERK phosphorylation by everolimus versus rapamycin^44^. Furthermore, different cell types exhibit specific levels of basal autophagy and thresholds to respond to autophagy triggers, resulting in different magnitudes of autophagy activation, which further contributes to the cell-specific differences in response to drug treatment^26^. Thus, our findings further underscore the importance of analyzing drug effects on multiple primary HIV-1 cellular targets, and on utilizing experimental infection models that more closely reiterate cellular diversity and relevant microenvironments, such as tissue biopsies or human organoid technology, for antiviral drug screening ex vivo^45^.

Notably, the antiviral effect of the tested autophagy drugs was corroborated in relevant HIV-1 mucosal target tissues (Figs. 1E, 4B–G), from which limited cells are available. Prophylactic treatment with everolimus restored HIV-1 restriction in emigrated vaginal LCs ex vivo (Fig. 1E). Furthermore, prophylactic or therapeutic treatment with everolimus or rapamycin prevented HIV-1 acquisition (Fig. 4C) and controlled HIV-1 replication in ongoing viral infection of intestinal CD4^+^ T cells (Fig. 4E). Although prophylactic or therapeutic treatment with carbamazepine reduced HIV-1 replication in skin-derived CD4^+^ T cells (Figs. 2F, 3E), carbamazepine did not have a significant effect on HIV-1 replication in intestinal CD4^+^ T cells (Fig. 4C,E). These data were mirrored in experiments assessing extracellular virus production by prophylactically- or therapeutically-treated intestinal LPLs (Fig. 4F,G).

Repurposing of clinically approved drugs for use as antiviral host-directed therapies reduces both the high cost required for developing novel drugs, as well as the time necessary to bring those drugs through clinical trials. Furthermore, combinatory use of both host-directed and direct-acting antivirals may potentiate the efficacy of direct-acting antivirals by optimizing host protective immune defenses, thereby accelerating treatment efficacy and reducing likelihood of antiviral resistance, as has already been demonstrated in tuberculosis treatment regimens^20^. Notably, HIV-1 infected individuals who received the autophagy drug rapamycin after liver transplantation showed significantly better control of HIV-1 replication versus patients receiving an alternative immunosuppressant^46^. The potential of autophagy-based therapeutics has also been illustrated in ex vivo PBMC and CD4^+^ T cells derived from HIV-infected individuals, where treatment with rapamycin or DIABLO/SMAC mimetics respectively reduced HIV-1 replication, or selectively induced killing of latently infected CD4^+^ T cells^27,47^. These data are further supported by in vitro studies demonstrating that treatment with autophagy-enhancing peptides or vitamin D, also shown to promote autophagy, inhibits HIV-1 infection in human macrophages^48,49^. More recently, trehalose, another autophagy inducer, has also been shown to not only reverse the HIV-1-mediated autophagy block in U937-derived macrophages and human monocyte-derived macrophages, but also to promote intracellular viral degradation in macrophages and CD4^+^ T cells^50,51^. Altogether, these studies corroborate that autophagy is a relevant target for preventing and intervening in HIV-1 infection.

Our findings describe the potential of repurposing clinically approved, autophagy-targeting drugs for both limiting cell-to-cell transmission and reducing ongoing HIV-1 replication in tissue-derived cellular reservoirs. Further studies are required to elucidate optimized combinatory strategies of host-directed therapies and direct-acting antivirals, taking into account drug pharmacokinetic interactions and stability^52,53^. In addition, as autophagy pathways impact inflammation, it would be interesting to investigate whether autophagy-based therapies could additionally mitigate immune activation, which not only undermines PrEP effectiveness but also contributes to the development of long-term comorbidities such as atherosclerosis and neurodegenerative diseases in treated HIV-1 patients^10,15^.

Hence, these results underscore host autophagy as a relevant emerging target for HIV-1 therapies. Furthermore, our novel HIV-1 infection model provides a suitable scientific framework for screening of additional host-directed and combinatory HIV-1 therapies ex vivo.

## Methods

### Ethics statement

Human skin and vaginal tissues tissue were obtained with approval of the Medical Ethics Review Committee of the Amsterdam University Medical Centers (Amsterdam UMC), Amsterdam, the Netherlands. Skin and vaginal tissues were handled in accordance with the relevant guidelines and regulations, as stated in the Amsterdam UMC Research Code. Use of these tissues is not subjected to informed consent according to the Medical Research Involving Human Subjects Act and the Medical Ethics Review Committee of Amsterdam UMC. Buffy coats derived from blood donations (Sanquin blood bank, the Netherlands) were obtained with approval of the Medical Ethics Review Committee of the Amsterdam UMC. Buffy coats were handled in accordance with the relevant guidelines and regulations, as stated in the Amsterdam UMC Research Code. Use of buffy coats is not subjected to informed consent according to the Medical Research Involving Human Subjects Act and the Medical Ethics Review Committee of Amsterdam UMC. Human fetal intestinal tissue (gestational age 18–20 weeks) was obtained by the HIS Mouse Facility of the Amsterdam UMC from the Bloemenhove clinic (Heemstede, the Netherlands), with a written informed consent obtained from all donors for the use of the material for research purposes. These informed consents are kept together with the medical record of the donor by the clinic. Tissues were obtained with approval of the ethical committee of the Amsterdam UMC, together with approval of the experimental procedures by the HIS Mouse Facility (Amsterdam UMC). All methods were performed in accordance with the relevant guidelines and regulations, as stated in the Amsterdam UMC Research Code.

### Antibodies and reagents

The following antibodies were used: anti-human CD1a (HI149-APC; BD Pharmigen), CD3 (UCTH1-APC-FIRE750; Biolegend), anti-human CD8a (HIT8a-PerCP-Cy5.5), anti-human CD14 (HCD14-PE-Dazzle594), anti-human CD11c (B-ly6-PE-Cy7), anti-HIV-1 capsid protein p24 (KC57-PE; Beckman Coulter), anti-human LC3 (4E12; MBL Life Science), goat anti-mouse IgG1 (AF488; Invitrogen A-21121).

The following reagents were used: bafilomycin A1 (Invivogen), carbamazepine (Tocris), everolimus (Invivogen), rapamycin (Invivogen), Zidovudine (AZT; NIH AIDS Reagent Program, Division of AIDS, NIAID, NIH), paraformaldehyde (8%, Electron Microscopy Sciences, Aurion), dispase II (Roche Diagnostics), ethylenediaminetetraacetic acid (EDTA; Sigma-Aldrich), 2 mM 1,4-dithiothreitol (DTT; Sigma-Aldrich), Collagenase D (Roche), DNAse type I (Worthington Biochemical Corporation).

### Cell lines

U87 cell lines stably expressing CD4 and wild-type CCR5 co-receptor were obtained through the NIH AIDS Reagent Program, Division of AIDS, NIAID, NIH: U87 CD4^+^CCR5^+^ cells from Deng and Littman^54^. Autophagy reporter cells were generated via retroviral transduction of U87.CD4.CCR5 with pBABE-mCherry-GFP-LC3 (Addgene 22418; gift from Prof. Jayanta Debnath^55^), as described previously^35^, hereafter referred to as U87.LC3-mCherry-GFP cells. U87.CD4.CCR5 parental and U87.LC3-mCherry-GFP cells were maintained in Iscoves Modified Dulbecco's Medium (IMDM, Thermo Fischer Scientific, USA) supplemented with 10% FCS and penicillin/streptomycin (10 U/ml and 10 μg/ml, respectively; Invitrogen).

### HIV-1

HIV-1 NL4.3BaL, HIV Gag-iGFP, and THRO were produced as previously described^12,13^. HIV Gag-iGFP plasmid was obtained through the NIH AIDS Reagent Program, Division of AIDS, NIAID, NIH: HIV Gag-iGFP (Cat# 12457), courtesy of Dr. Benjamin Chen^56–58^. HIV-1 THRO plasmid was obtained through the NIH AIDS Reagent Program, Division of AIDS, NIAID, NIH: p.THRO.c/2626, Panel of full-length transmitted/founder (T/F) HIV-1 Infectious Molecular Clones (Cat #11919), courtesy of Dr. John Kappes and Dr. Christina Ochsenbauer^59,60^. Plasmids were amplified by transformation into STBL3 *E. coli* bacteria (Invitrogen), and 293T cells were subsequently transfected with proviral plasmids. Viruses were harvested at day 2 and 3. The p24 content of all produced viruses was quantified by p24 antigen ELISA (Perkin Elmer Life Sciences). TCID50 was determined using TZM-bl indicator cells (John C. Kappes, Xiaoyun Wu, Birmingham, Alabama, USA and Tranzyme Inc., the NIH AIDS Reagent Program, division of AIDS, NIAID) as previously described^12^.

### HIV-1 tissue infection model for drug screening

We developed a novel HIV-1 human tissue infection model for analysis of multiple immune cell types that are targets for HIV-1. Human skin tissues were obtained from healthy donors undergoing corrective breast or abdominal surgeries as previously described^12,13^, and with ethical approval as described above. Skin sheets approximately 1.2 mm thick, containing epidermis and dermis, were obtained using a dermatome (Zimmer), and then cut it into uniform circles of 12 mm diameter using a customized punch biopsy instrument, for culture in a 24-well plate in supplemented IMDM.

In the prophylactic treatment model, biopsies were treated with carbamazepine (100 μM), everolimus (5 nM), or rapamycin (100 nM) for 15 h, or left untreated, and subsequently infected with HIV-1 NL4.3-BaL (100 μl/biopsy, TCID50 of 56 × 10^3^, determined in TZM-Bl cells). In the therapeutic treatment model, biopsies were first infected with HIV-1 NL4.3-BaL (100 μl/biopsy, TCID50 of 56 × 10^3^, determined in TZM-Bl cells), or left uninfected, and 15 h later treated with the autophagy drugs. Drug concentrations and incubation times were optimized based on cell viability (Figure S1, Supplementary Information) and tissue thickness. To confirm productive HIV-1 infection of tissue-derived cells, tissue biopsies were treated with HIV-1 replication inhibitor AZT (zidovudine, 20 μM).

After 3 days of culture, emigrated tissue cells were washed and replated for an additional 72 h. Emigrated cells were then harvested and HIV-1 infection of different cell types and subsets was quantified using multiparameter flow cytometry in line with^61^, as detailed in Fig. S4, Supplementary Information. HIV-1 infection per donor was quantified as the percent of CD11c^+^ DCs or CD4^+^ T cells stained positive for HIV-1 capsid protein p24. Infection of CD11c^+^ DCs was defined as CD3^−^CD11c^+^p24^+^ cells. HIV-1 infection of CD4^+^ T cells was defined as CD3^+^CD11c^−^CD14^−^CD1a^−^CD8^−^p24^+^ cells. A detailed overview of this model is available in Figure S3A,C, Supplementary Information.

### Epidermal explant HIV-1 infection model

Human skin tissues were obtained from healthy donors as described above. Skin sheets approximately 0.3 mm thick were obtained using a dermatome (Zimmer), and then incubated with dispase II (1 mg/ml, Roche Diagnostics) in IMDM supplemented with 10% FCS, gentamicin (20 μg/ml, Centrafarm, Netherlands), penicillin/streptomycin (10 U/ml and 10 μg/ml, respectively; Invitrogen) for either 1.5 h at 37 °C or overnight at 4 °C. Epidermis was separated from dermis by hand using sterilized metal tweezers, washed in PBS followed by IMDM, and then cut into uniform circles of 12 mm diameter using a customized punch biopsy instrument.

Epidermal sheets were subsequently pre-incubated with 100 μM carbamazepine, 30 nM everolimus, or 100 nM rapamycin for 2 h before infection with HIV-1 NL4.3BaL (80 μl/biopsy, TCID50 of 56 × 10^3^, determined in TZM-Bl cells) or transmitted/founder HIV-1 strain THRO (80 μl/biopsy, TCID50 of 125 × 10^3^, determined in TZM-Bl cells). A detailed overview of this model is available in Figure S2A, Supplementary Information.

### Vaginal LC isolation and HIV-1 infection

Vaginal mucosa was incubated with dispase II (3 mg/mL, Roche Diagnostics) in IMDM, to permit separation of mucosa from submucosa. Mucosal sheets were subsequently cultured in fully supplemented IMDM until the tissue disintegrated. Further vaginal LC purification was performed using a Ficoll gradient and CD1a microbeads (Miltenyi Biotec). Isolated LCs were routinely > 80% pure and expressed high levels of Langerin and CD1a. CD1a^+^ vaginal LCs were pre-incubated with 30 nM everolimus for 2 h before infection with HIV-1 NL4.3BaL at MOI = 0.09.

### HIV-infection of human intestinal lymphocytes

Intestinal LPLs were obtained as previously described^40^. Briefly, fetal intestines were cut open longitudinally and cleaned by washing extensively in PBS. To detach the epithelial layer, intestinal tissues were then cut into 0.5 × 0.5 cm segments and incubated in PBS containing 5 mM EDTA (Sigma-Aldrich), 2 mM DTT (Sigma-Aldrich), penicillin/streptomycin (10 U/ml and 10 μg/ml, respectively; Invitrogen), and 1% fetal calf serum (FCS), in a shaking water bath at 4 °C for 2 × 20 min. The intestinal tissue, now lacking the epithelial layer, was minced and digested for 2 × 30 min at  37 °C with IMDM supplemented with 1 mg/ml (0.15 U/mg) Collagenase D (Roche), penicillin/streptomycin (10 U/ml and 10 μg/ml, respectively; Invitrogen), 1% FCS, and 1000 U/ml DNAse type I (Worthington Biochemical Corporation). The supernatant containing the cells was filtered through a 70 μm strainer (Falcon, Corning), washed, and re-filtered to obtain a single cell solution. After isolation, the number of viable cells was counted using Trypan blue (Sigma-Aldrich). LPLs were cultured in IMDM supplemented with 10% FCS, penicillin/streptomycin (10 U/ml and 10 μg/ml, respectively; Invitrogen), and 50 U/mL IL-2 (Miltenyi).

Freshly isolated LPLs were utilized in HIV-1 infection experiments and cellular composition of the sample was determined by flow cytometry analysis. The major cell type in LPL samples was CD3^+^CD4^+^ T cells, and CD11c^+^ DCs were negligible (Fig. S7A,D, Supplementary Information). Drug concentrations and incubation times were optimized based on cell survival and viability (Figure S7B,C, Supplementary Information). For the prophylactic treatment model, LPLs were pre-incubated with 100 μM carbamazepine, 5 nM everolimus, or 100 nM rapamycin, or left untreated, for 15 h before infection with HIV-1 NL4.3BaL at MOI = 0.02 in a 96-well plate. Alternatively, for the therapeutic treatment model, LPLs were infected with HIV-1 NL4.3BaL at MOI = 0.02 for 15 h prior to treatment with 100 μM carbamazepine, 5 nM everolimus, or 100 nM rapamycin. As a control for replicative infection of LPLs, AZT (20 μM) was administered. A detailed overview of this model is available in Figure S7E,F (Supplementary Information)**.**

### HIV-1 transmission using U87.CD4.CCR5 cells

After 72 h of infection, emigrated epidermal LCs, vaginal LCs, or LPLs were extensively washed to remove unbound virus, and co-cultured with the HIV-1 permissive U87.CD4.CCR5 cell line for an additional 72 h. HIV-1 infection of U87.CD4.CCR5 cells is representative of HIV-1 transmission by LCs and T cells, and was determined by intracellular p24 staining by flow cytometer. LC marker CD1a and T cell marker CD3 were respectively used to exclude CD1a^+^ LCs and LC-U87 conjugates, and CD3^+^ LPLs and LPL-U87 conjugates from analysis in the co-culture.

### Extracellular HIV-1 release using U87.CD4.CCR5 cells

To further confirm productive HIV-1 infection of skin-derived cells, the supernatants from prophylactically or therapeutically treated and HIV-1 infected emigrated cells was harvested 120 h after extensively washing to remove input virus, and transferred onto HIV-1 permissive U87.CD4.CCR5 cells for a period of 72 h (schematic representation in Fig. S3B,D, Supplementary Information). Likewise, to confirm productive HIV-1 infection of intestinal LPLs, the supernatants from prophylactically or therapeutically treated and HIV-1 infected LPLs was harvested 24 h after extensively washing to remove input virus, and transferred onto HIV-1 permissive U87.CD4.CCR5 cells for a period of 72 h. HIV-1 infection of U87.CD4.CCR5 cells was representative of production of infectious extracellular HIV-1 virus by emigrated tissue-derived cells or LPLs respectively, and was determined by intracellular p24 staining by flow cytometer.

### Dendritic cell isolation and HIV-1 infection

Monocyte-derived DCs were generated as previously described^33^. Briefly, PBMCs were isolated from buffy coats of healthy donors (Sanquin) using a Lymphoprep (Axis-Shield) gradient. Subsequently, a Percoll (Amersham Biosciences) gradient step was used to enrich for monocytes. Monocytes were then differentiated into immature DCs over 6 days in RPMI 1640 containing 10% FCS, penicillin/streptomycin (10 U/ml and 10 μg/ml, respectively; Invitrogen), and 2 mM l-glutamine (Lonza), and supplemented with 500 U/ml IL-4 (Invitrogen) and 800 U/ml GM-CSF (Invitrogen). This protocol consistently generates high-purity immature DCs expressing typical markers DC-SIGN, CD11c, and CD1a, as measured by flow cytometry^38^. DCs were infected with HIV-1 NL4.3BaL at MOI = 0.02 for 15 h prior to treatment with 5 nM everolimus for 6 days. As a control for productive infection of DCs, AZT was administered. After 6 days of HIV-1 infection, cells were washed, harvested, and stained for intracellular HIV-1 capsid protein p24. HIV-1 infection of DCs was determined by p24 intracellular staining by flow cytometer.

### Measurement of autophagy flux

U87.LC3-mCherry-GFP cells were treated with carbamazepine (1000 μM; Tocris), everolimus (30 nM; Invivogen), or rapamycin (100 nM; Invivogen), or left untreated for 24 h. LC3 is a canonical marker of autophagy. LC3 coats autophagosomal membranes (termed LC3-II), and during autophagy flux, luminal LC3-II within autophagosomes is degraded due to acidic pH upon autophagosome-lysosome fusion^35^. The mCherry tag is acid-insensitive while the GFP tag is acid-sensitive, and GFP signal is quenched upon autophagosome acidification. Reduction in GFP signal measured by flow cytometer analysis is thereby representative of induction of autophagy flux^35^. Loss of GFP signal was determined in live, singlet, mCherry^+^GFP^+^ U87s. Bafilomycin A1 halts autophagosome-lysosome fusion, therefore a control gate to determine autophagy flux upon drug treatment was set in bafilomycin A1-treated (100 nM) cells, as previously described^35,62^.

Autophagy flux was also measured in tissue-derived cells. Uniform human skin biopsies containing epidermis and dermis were obtained as described above, and cultured in a 24-well plate in supplemented IMDM, with carbamazepine (100 μM), everolimus (5 nM), or rapamycin (100 nM), or left untreated. After 15 h incubation with the autophagy drugs, skin biopsies were subsequently treated with lysosomal inhibitor bafilomycin A1 (200 nM). 24 h later, emigrated tissue cells were harvested and intracellular LC3-II accumulation in different cell types was quantified using multiparameter flow cytometry, as previously described^21,36,37^. Finally, autophagy induction was additionally measured in human lamina propria lymphocytes. Intestinal LPLs were obtained as described above, and cultured in IMDM supplemented with 10% FCS, penicillin/streptomycin (10 U/ml and 10 μg/ml, respectively; Invitrogen), and 50 U/mL IL-2, and with or without bafilomycin A1 (50 nM). 2 h later, LPLs were treated with carbamazepine (100 μM), everolimus (5 nM), or rapamycin (100 nM), or left untreated. After 15 h incubation with autophagy drugs, LPLs were harvested and intracellular LC3-II accumulation in different cell types was quantified using multiparameter flow cytometry, as previously described^21,36,37^.

### Data analysis and statistics

GFP and mCherry fluorescence was measured by flow cytometry on a FACS LSR-FORTESSA (BD Biosciences), and HIV-1 transmission and infection experiments were measured on a FACSCanto II (BD Biosciences). Data was analyzed using FlowJo, LLC, version 10 (Treestar) and GraphPad Prism 8. A two-tailed, parametric Student's *t* test was performed for paired observations (Fig. 1B). For data shown in relative, data was normalised to untreated, HIV-1 infected samples (set at 1). A one-sample *t* test was then utilized to compare fold changes in experimental conditions (drug-treated-HIV-1 infected samples) to the hypothetical population mean of 1, in line with^63^ (Figs. 1C, 2D,F, 3D,E, 4C,E; S6B in Supplementary Information). Data in which donors were subjected to repeated measures was analysed using mixed-effects analysis of variance (ANOVA; Fig. S1 of supplementary information). Statistical analyses were performed using GraphPad Prism 8 software and significance was set at *P < 0.05, **P < 0.01, ***P < 0.001.

## Supplementary Information

Supplementary Information.

## Data Availability

The majority of data generated or analysed during this study are included in this published article and its Supplementary Information files. Additional datasets generated and/or analysed during the current study are available from the corresponding author upon reasonable request.
