# Supplementary material for: Autophagy-enhancing drugs limit mucosal HIV-1 acquisition and suppress viral replication ex vivo
Source: Sci Rep. 2021 Feb 26;11:4767. doi: 10.1038/s41598-021-84081-4 (PMC7910550; doi:10.1038/s41598-021-84081-4)
Supplement: Supplementary file 1 — Supplementary Information. [file 41598_2021_84081_MOESM1_ESM.pdf]

Autophagy-enhancing drugs limit mucosal HIV-1 acquisition and suppress viral replication *ex vivo*

Alexandra P.M. Cloherty, Nienke H. van Teijlingen, Tracy-Jane T.H.D. Eisden,
John L. van Hamme, Anusca G. Rader, Teunis B.H. Geijtenbeek, Renée R.C.E. Schreurs,
Carla M.S. Ribeiro

Extended Data

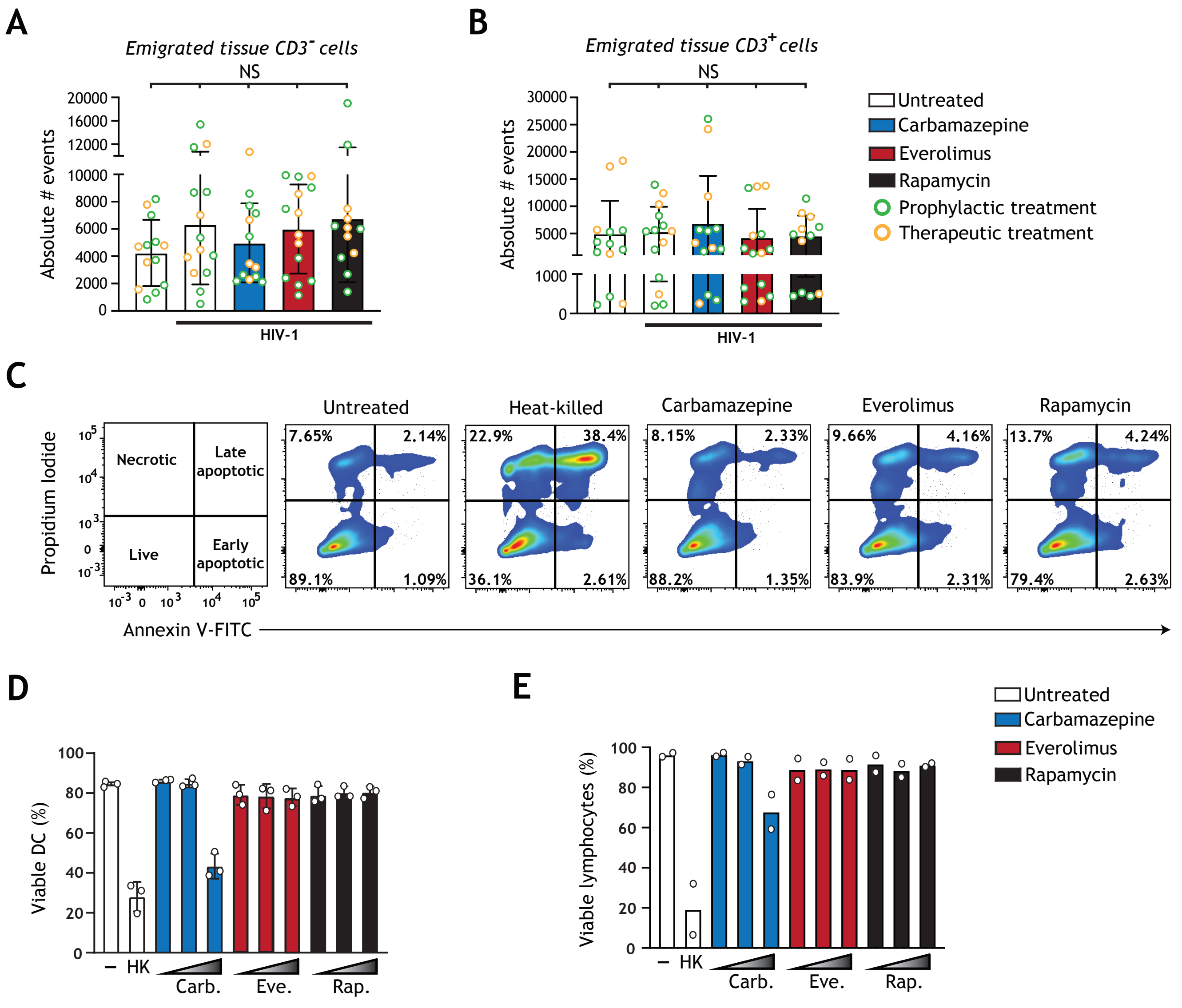

Supplementary Information Figure S1. Validation of optimized concentrations of autophagy drugs.
(A-B) Biopsies including epithelium and subepithelium were taken from human skin tissue and either prophylactically (green open circles) or therapeutically (orange open circles) treated with carbamazepine (100µM), everolimus (5nM), or rapamycin (100nM), or left untreated, followed by HIV-1 infection for 72 hours. Emigrated cells were harvested, stained (as depicted in **Fig. 2B**), and all events were acquired by flow cytometer. The absolute number of emigrated CD3- **(A)** and CD3+ **(B)** cells across the different experiments was determined. The different drug treatments, HIV-1 innoculation or prophylactic/therapeutic administration did not significantly change the quantity of emigrated viable cells from tissue biopsies (n.s., non-significant; mixed effects ANOVA). Open circles represent the mean of duplicates from independent donors, n=13-14. **(C-E)** Human blood-derived DCs and lymphocytes were treated with carbamazepine (20µM, 100µM, 500µM), everolimus (2nM, 5nM, 10nM), rapamycin (20nM, 100nM, 500nM), or medium control for 48h, or heat-killed by exposing to 56°C for 10 minutes. Blood-derived cell types were subsequently stained using an annexin-V-FITC and PI (eBioscience) and measured by flow cytometry. FITC-conjugated annexin-V binds to phosphatidylserine, a phospholipid that is externalized relatively early during apoptosis, and PI is a marker for loss of membrane integrity and necrosis. Therefore, viable cells are annexin-V/FITC- PI- . Gates were set based on cell populations left untreated or heat-killed for 10 minutes at 56°C. **(C)** Representative flow cytometry plots of n=3 independent experiments, showing both DCs and lymphocytes treated with 100nM carbamazepine, 5nM everolimus, or 100nM rapamycin. **(D,E)** Proportion of viable (annexin-V/FITC- PI-) DCs **(D)** and lymphocytes **(E)** following heat-killing or treatment with concentration ranges of carbamazepine, everolimus, or rapamycin. Only heat-killing or treatment with 500nM carbamazepine significantly reduced DC **(D)** or lymphocyte **(E)** viability. **(C-E)** Selected concentrations for tissue infection experiments were 100µM carbamazepine, 5-30nM everolimus, and 100nM rapamycin. Data are mean ± SE of n=2-3 donors measured in duplicate. Open circles represent the mean of duplicates from independent donors.

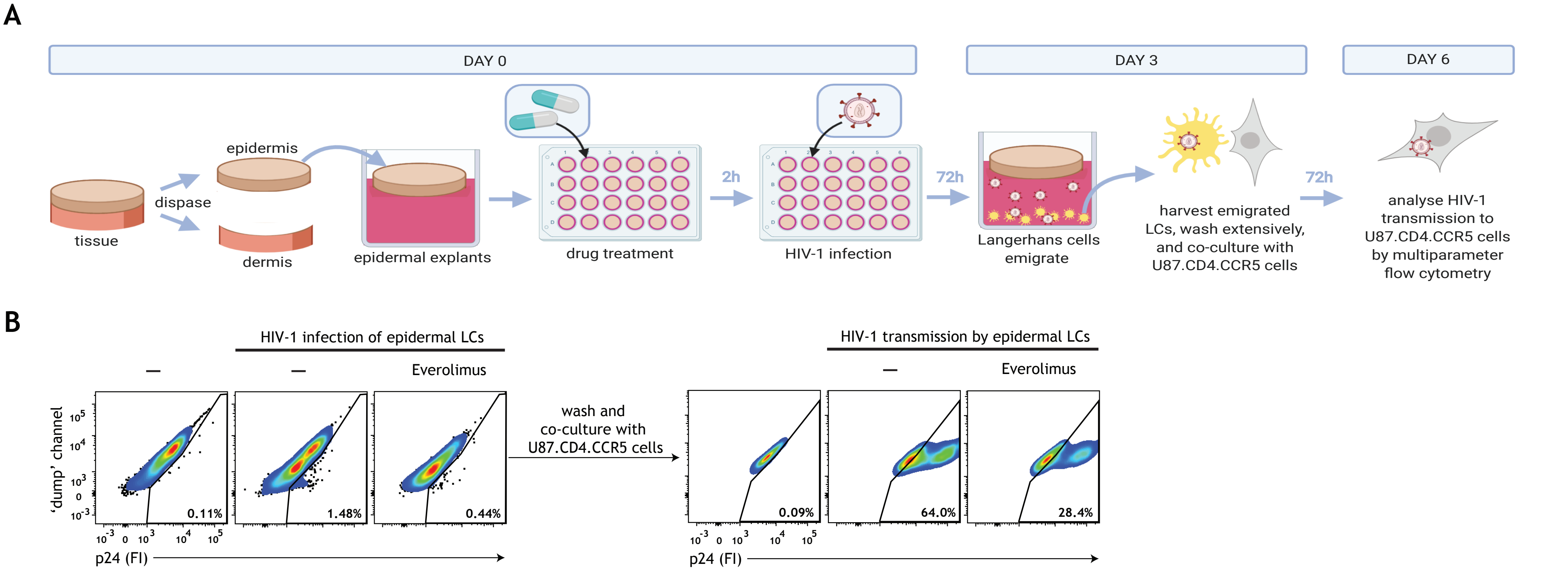

Supplementary Information Figure S2. HIV-1 infection of primary activated human Langerhans cells and HIV-1 transmission to U87.CD4.CCR5 is limited by everolimus treatment. **(A)** Schematic representation of the epidermal explant HIV-1 transmission model. Human skin tissues from healthy donors of approximately 0.3 mm thickness were obtained using a dermatome. The tissue was then incubated with dispase II for either 1.5 h at 37°C or overnight at 4°C. Epidermis was separated from dermis by hand, washed, and then uniform circle biopsies of 12 mm diameter were taken using a customized punch biopsy instrument. Epidermal biopsies were subsequently pre-incubated with selected autophagy drugs for 2 hours before infection with HIV-1 NL4.3BaL or transmitted/founder HIV-1 strain THRO (day 0). After 72 hours of infection (day 3), emigrated epidermal LCs were extensively washed to remove unbound virus, and co-cultured with the HIV-1 permissive U87.CD4.CCR5 cell line for an additional 72 hours (to day 6). HIV-1 transmission to the U87.CD4.CCR5 cell line was determined by intracellular p24 staining by flow cytometer. **(B)** Human epidermal biopsies were prophylactically treated with 30nM everolimus, or left untreated, and subsequently infected with HIV-1 NL4.3 BaL for 72 hours. Emigrated LCs were extensively washed, and either replated for an additional 72 hours, or co-cultured with U87.CD4.CCR5 cells for 72 hours. HIV-1 infection of activated emigrated LCs was determined by intracellular p24 staining by flow cytometer, and was approximately 1-2%, in accordance with previous reports [12,21]. HIV-1 transmission by LCs was assessed in LC-U87.CD4.CCR5 co-culture for 72 hours, determined by intracellular p24 staining by flow cytometer, which provided a clear readout for HIV-1 transmission by LCs, in accordance with [12-14, 21]. LC-marker CD1a was used to exclude single LCs and LC-U87 conjugates from analysis. Representative flow cytometry plots of n=2 donors measured in duplicate demonstrate a reduction in HIV-1 infection of LCs and transmission by LCs upon everolimus treatment.

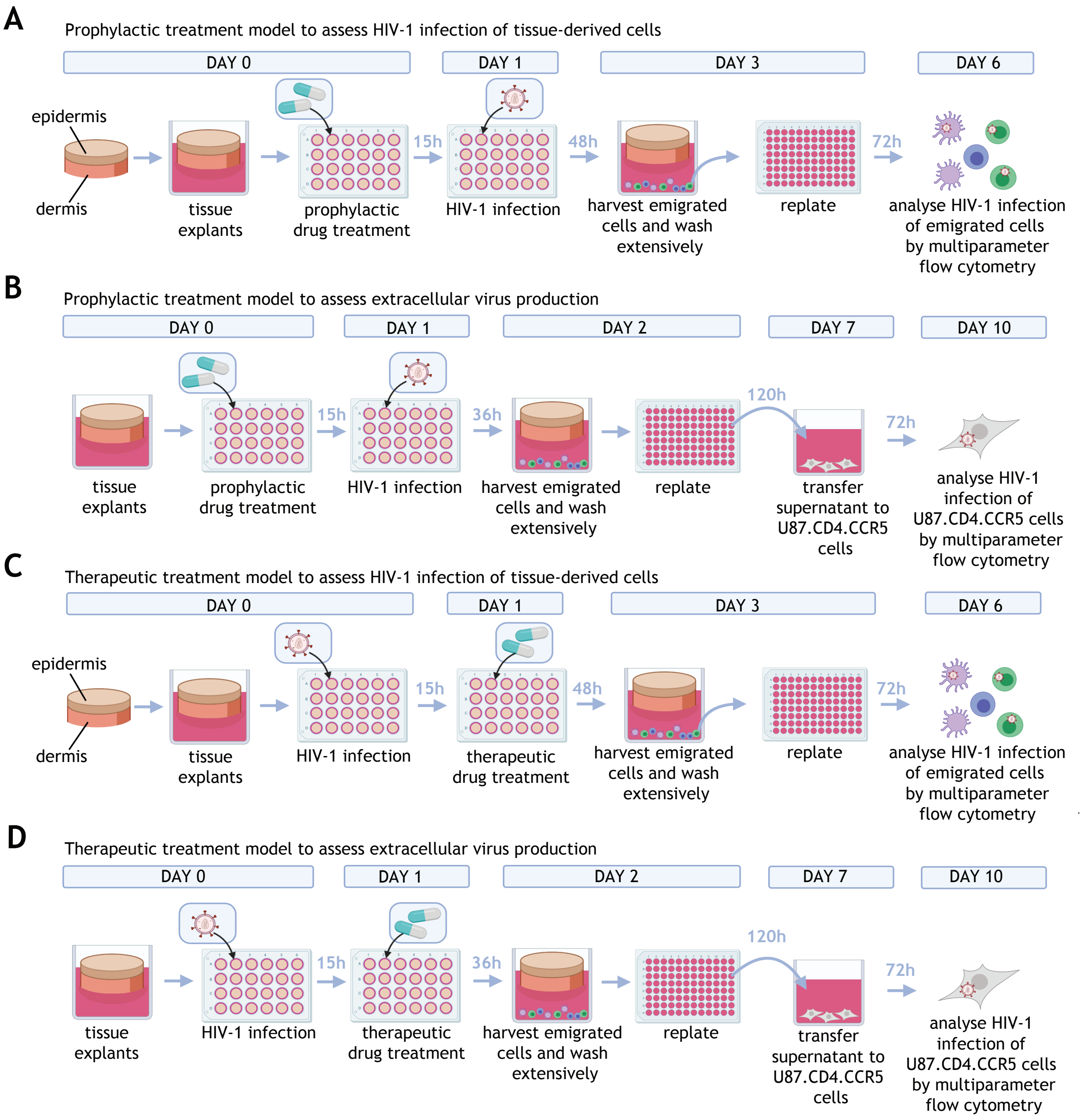

Supplementary Information Figure S3. Detailed graphical representation of our novel HIV-1 infection model for drug screening. **(A)** To assess HIV-1 infection of tissue-derived cells in a prophylactic treatment setting, human skin tissues from healthy donors of approximately 1.2 mm thick, containing both epidermis and dermis, were obtained using a dermatome. Uniform circle biopsies of 12mm diameter were then obtained using a customized punch biopsy instrument, for culture in a 24-well plate with selected autophagy drugs (day 0). 15 hours later (day 1) tissue biopsies were infected with HIV-1 NL4.3-BaL. After 72 hours of culture (day 3), emigrated tissue cells were washed and replated for an additional 72 hours (day 6). HIV-1 infection of different cell types and subsets was quantified using multiparameter flow cytometry as detailed in **Figure S4**, Supplementary Information. **(B)** To assess extracellular HIV-1 production by tissue-derived cells in a prophylactic treatment setting, human skin biopsies were prophylactically treated prior to HIV-1 NL4.3-BaL infection as in **(A)**. 24 hours after infection (day 3), emigrated tissue-derived cells were extensively washed to remove input virus, and replated in new medium in a 96-well plate. 120 hours after replating (day 7), supernatant from infected tissue-derived cells was collected and incubated with U87.CD4.CCR5 cells for 72 hours (day 10), to confirm production of extracellular virus. HIV-1 infection of U87.CD4.CCR5 cells was determined by intracellular p24 staining by flow cytometer.
(C) In our therapeutic HIV-1 tissue infection model for drug screening, uniform human skin biopsies containing epidermis and dermis were obtained as in **(A)**, and infected with HIV-1 NL4.3-BaL (day 0). 15 hours later (day 1) tissue biopsies were treated with selected autophagy drugs. After 72 hours of culture (day 3), emigrated tissue cells were washed and replated for an additional 72 hours (day 6). HIV-1 infection of different cell types and subsets was quantified using multiparameter flow cytometry as detailed in **Figure S4**, Supplementary Information. **(D)** To assess extracellular HIV-1 production by tissue-derived cells in a therapeutic treatment setting, human skin biopsies were infected with HIV-1 NL4.3-BaL and subsequently therapeutically treated as in **(C)**. 24 hours after treatment (day 2), emigrated tissue-derived cells were extensively washed to remove input virus, and replated in new medium in a 96-well plate. 120 hours after replating (day 7), supernatant from infected tissue-derived cells was collected and incubated with U87.CD4.CCR5 cells for 72 hours (day 10), to confirm production of extracellular virus. HIV-1 infection of U87.CD4.CCR5 cells was determined by intracellular p24 staining by flow cytometer.

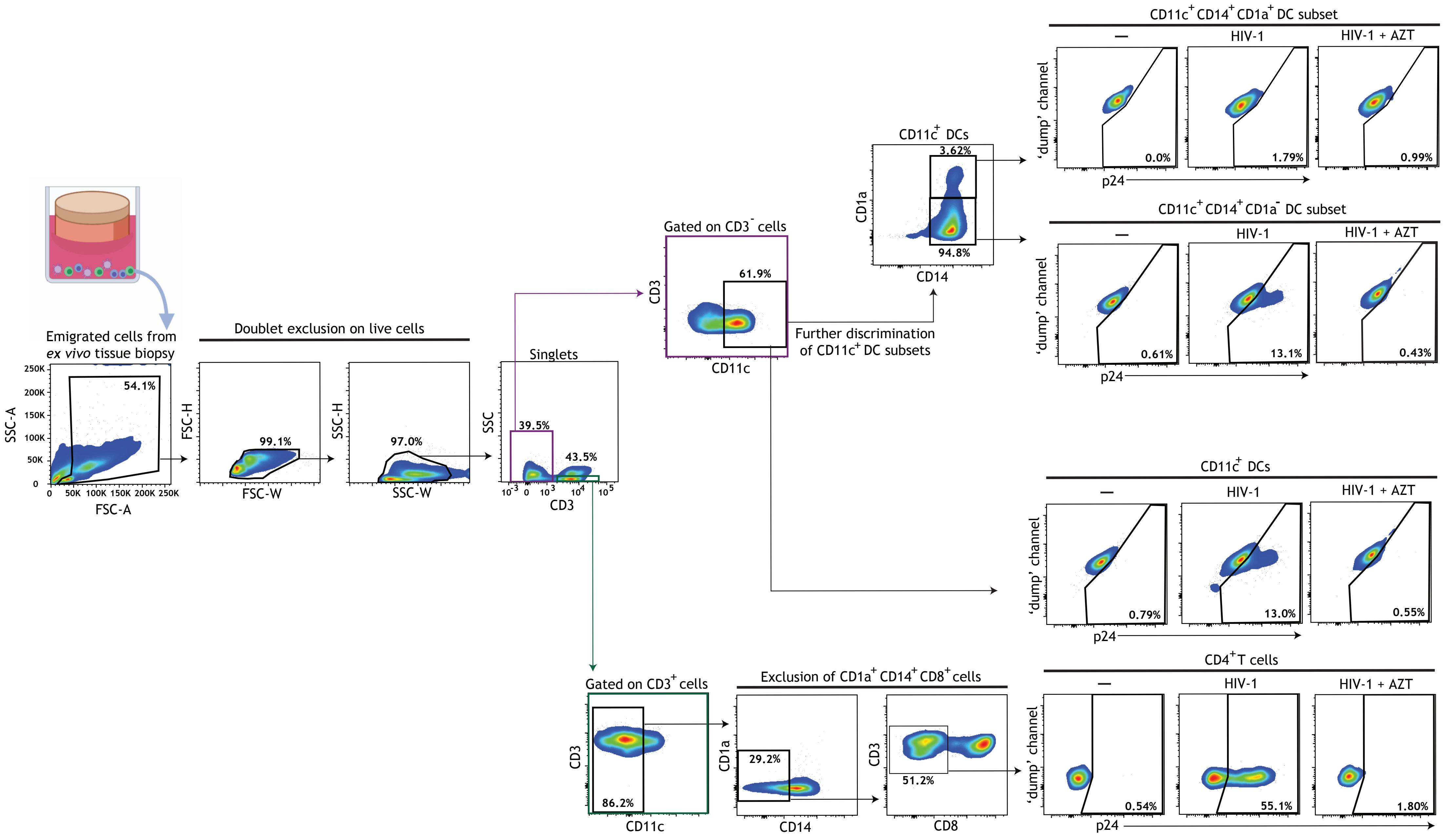

Emigrated cells from
ex vivo tissue biopsy
Doublet exclusion on live cells
Singlets
Gated on CD3- cells
CD11c+ DCs
CD11c+ CD14+ CD1a+ DC subset
CD11c+ CD14+ CD1a- DC subset
Further discrimination of CD11c+ DC subsets
Gated on CD3+ cells
Exclusion of CD1a+ CD14+ CD8+ cells
CD4+ T cells
HIV-1
HIV-1 + AZT
'dump' channel
p24
SSC-A
FSC-A
FSC-H
FSC-W
SSC-H
SSC-W
SSC
CD3
CD11c
CD1a
CD14
CD8
54.1%
99.1%
97.0%
39.5%
43.5%
61.9%
3.62%
94.8%
86.2%
29.2%
51.2%
0.0%
1.79%
0.99%
0.61%
13.1%
0.43%
0.79%
13.0%
0.55%
0.54%
55.1%
1.80%

Supplementary Information Figure S4. Gating strategy utilized to identify emigrated CD11c+ DC subsets and CD4+ T cells in HIV-1 tissue infection model. Biopsies including epithelium and subepithelium were taken from human skin tissue and cultured in a 24 well plate. Ex vivo tissue was prophylactically or therapeutically treated with carbamazepine (100μM), everolimus (5nM), or rapamycin (100nM), or left untreated, and infected with HIV-1 NL4.3BaL. HIV-1 infection of different cell types and subsets was quantified 6-7 days p.i. using multiparameter flow cytometry. Treatment of tissue biopsies with HIV-1 replication inhibitor AZT (zidovudine, 20uM) confirms productive HIV-1 infection of these cell types. Singlet, live cells were gated on CD3 expression. CD3- cells that were also CD11c+ were considered to be CD11c+ DCs. Emigrated CD11c+ DCs could be further discriminated based on CD1a and CD14 expression, in accordance with previous reports [7,59]. HIV-1 infection of different DC subsets was thereby represented by the following cell populations: CD3-CD11c+p24+ cells, CD11c+CD14+CD1a+p24+ cells, or CD11c+CD14+CD1a-p24+ cells. The CD11c+CD14+CD1a- subset, which made up the majority of measured cells, was productively infected, but the smaller CD11c+CD14+CD1a+ cell subset was not. Infection in the CD3-CD11c+ subset therefore primarily reflected infection of CD11c+CD14+CD1a- cells. CD3+ cells were analysed using a complementary strategy to eliminate CD11c+CD14+CD1a+ cells. CD4+ and CD8+ T cells were distinguished based on CD8 expression. HIV-1 infection of CD4+ T cells was thereby represented by CD3+CD11c-CD14-CD1a-CD8-p24+ cells.

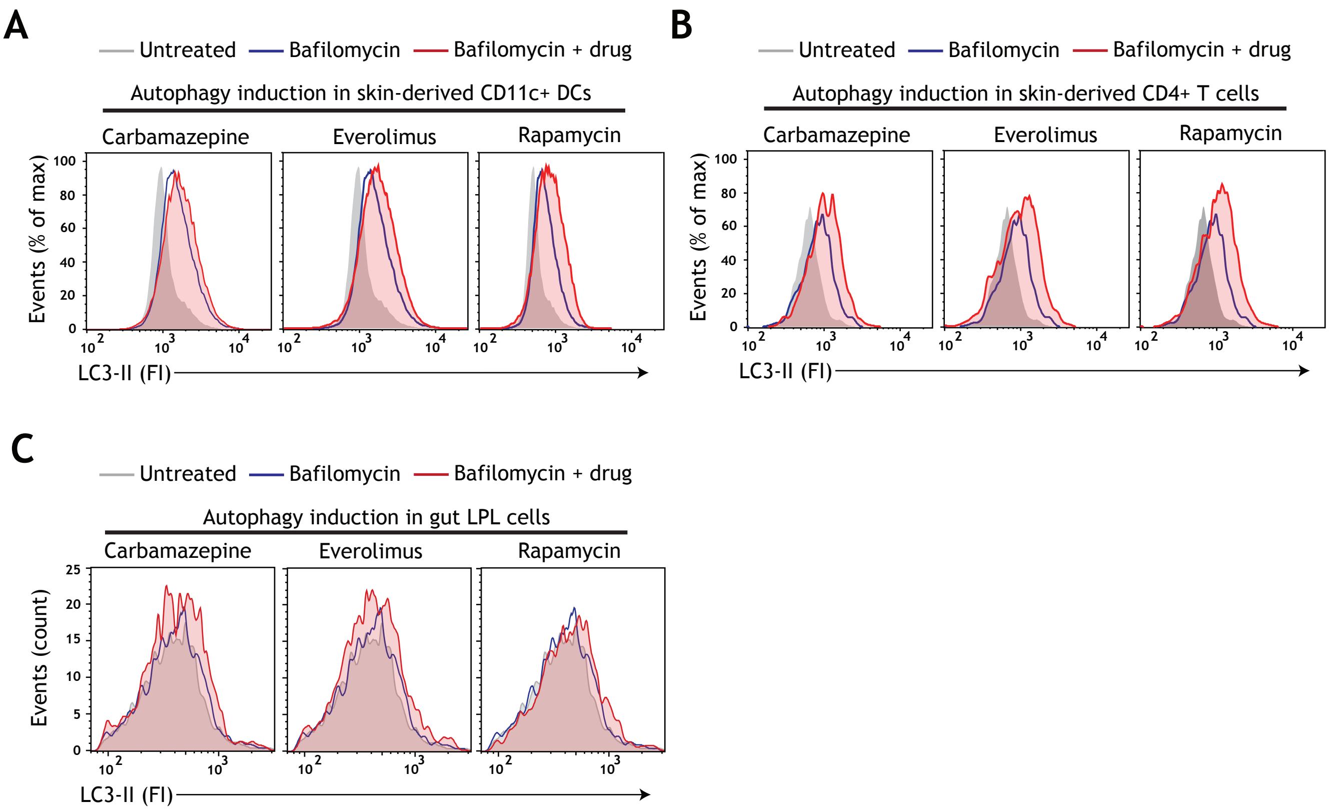

Supplementary Information Figure S5 Carbamazepine, everolimus, and rapamycin induce autophagy flux in tissue-derived CD11c+ DCs and CD4+ T cells. **(A,B)** Skin biopsies including epithelium and subepithelium were treated with autophagy drugs carbamazepine (100μM), everolimus (5nM), or rapamycin (100nM) for 15 hours, or left untreated. Subsequently, biopsies were treated with bafilomycin A1 (200nM) or left untreated for an additional 24 hours. **(C)** Gut LPLs were treated with lysosomal inhibitor bafilomycin A1 (50nM) for 2 hours, or left untreated. Subsequently, LPLs were treated with autophagy drugs carbamazepine (100μM), everolimus (5nM), or rapamycin (100nM) or left untreated for an additional 15 hours. Emigrated tissue cells **(A,B)** or gut LPLs **(C)** were harvested and autophagy induction in tissue-derived cells was determined by intracellular LC3-II levels by saponin extraction, as previously described [21,35-36], in combination with surface markers. A detailed gating strategy for distinguishing skin-derived CD11c+ DCs **(A)**, skin-derived CD4+ T cells **(B)** and gut T cells **(C)** can be found in **Figures S4, S7D**, Supplementary Information. Representative flow cytometry plots of n=2 donors measured in duplicate demonstrate that basal autophagy levels differ across cell types, and that autophagy drugs increase LC3-II levels at different maginitude in both skin- and gut-derived cells.

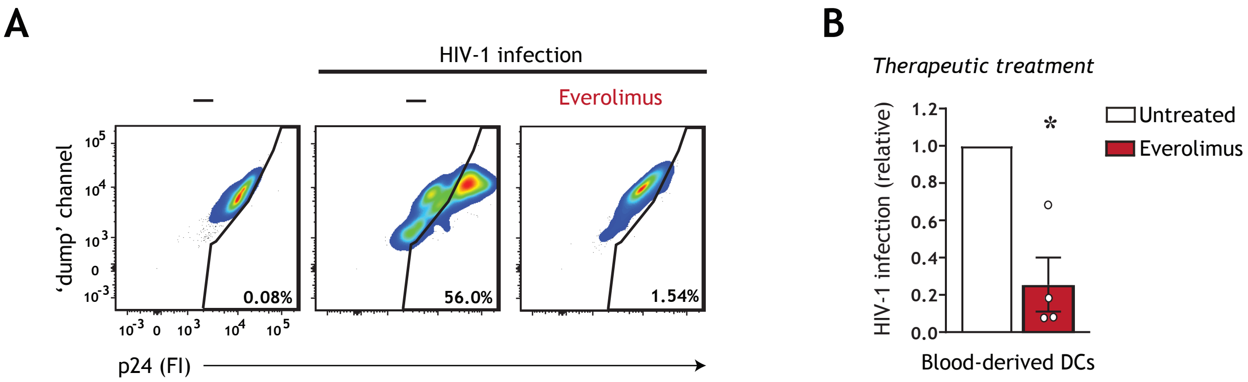

Supplementary Information Figure 6. Therapeutic treatment with everolimus reduces HIV-1 infection of human blood-derived DCs. **(A,B)** Monocyte-derived DCs were infected with HIV-1 NL4.3BaL for 15h, and subsequently treated with everolimus (5nM), or left untreated. HIV-1 infection of DCs was determined by intracellular p24 staining 6 days post infection by flow cytometer. Representative flow cytometry plots **(A)** and data are mean ± SE of n=4 donors **(B)**. Open circles represent the mean of duplicates from independent donors. *P < 0.05, one-sample t-test **(B)**.

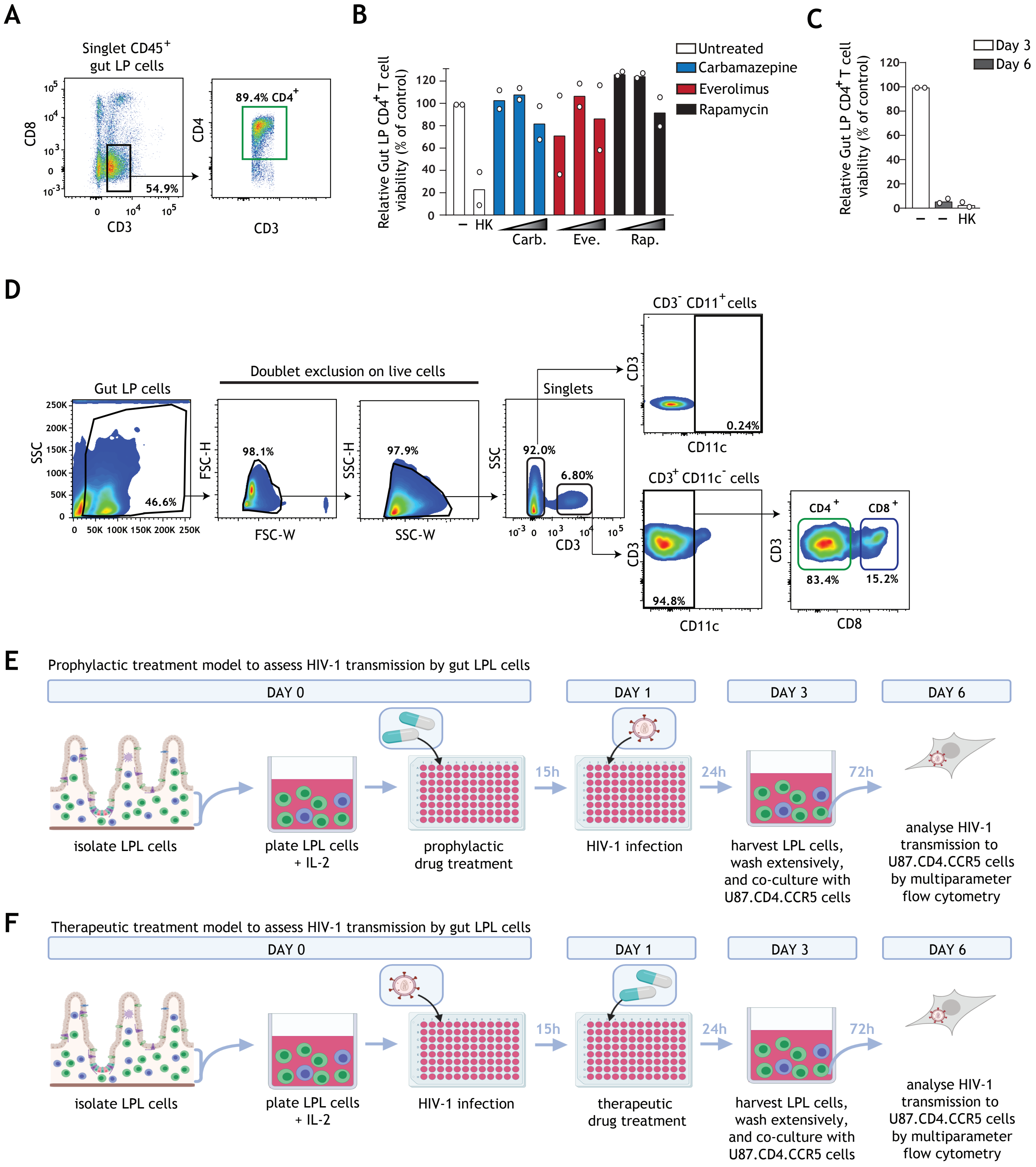
A
Singlet CD45+ gut LP cells
CD8
CD3
54.9%
89.4% CD4+
CD4
CD3
B
Relative Gut LP CD4+ T cell viability (% of control)
Untreated
Carbamazepine
Everolimus
Rapamycin
HK
Carb.
Eve.
Rap.
C
Relative Gut LP CD4+ T cell viability (% of control)
Day 3
Day 6
HK
D
Gut LP cells
SSC
46.6%
Doublet exclusion on live cells
FSC-H
98.1%
FSC-W
SSC-H
97.9%
SSC-W
Singlets
92.0%
6.80%
CD3
CD3- CD11+ cells
0.24%
CD11c
CD3+ CD11c- cells
94.8%
CD11c
CD4+
CD8+
83.4%
15.2%
CD8
E
Prophylactic treatment model to assess HIV-1 transmission by gut LPL cells
DAY 0
DAY 1
DAY 3
DAY 6
15h
24h
72h
isolate LPL cells
plate LPL cells + IL-2
prophylactic drug treatment
HIV-1 infection
harvest LPL cells, wash extensively, and co-culture with U87.CD4.CCR5 cells
analyse HIV-1 transmission to U87.CD4.CCR5 cells by multiparameter flow cytometry
F
Therapeutic treatment model to assess HIV-1 transmission by gut LPL cells
DAY 0
DAY 1
DAY 3
DAY 6
15h
24h
72h
isolate LPL cells
plate LPL cells + IL-2
HIV-1 infection
therapeutic drug treatment
harvest LPL cells, wash extensively, and co-culture with U87.CD4.CCR5 cells
analyse HIV-1 transmission to U87.CD4.CCR5 cells by multiparameter flow cytometry

Supplementary Information Figure S7. Viability, gating strategy, and model utilized to assess efficacy of prophylactic and therapeutic treatment with selected autophagy drugs on intestinal HIV-1 infection.
(A-F) Human fetal intestinal lamina propria lymphocytes (LPLs) were isolated as previously described [39]. **(A)** Singlet, live CD45+ LPLs were gated on CD3 and CD4 expression, confirming that CD3+CD8- LPLs represent primarily intestinal CD4+ T cells. **(B)** Untreated human gut LPLs were cultured for 72 hours or 6 days, or heat-killed by exposing to 56°C for 10 minutes. Cell viability was subsequently determined using the CellTitre-Glo® 3D Cell Viability Assay (Promega cat. #G9681) according to the manufacturer's instructions. Viability of gut LPLs cultured for 72 hours was set at 100%. Decreased viability of gut LPLs at day 6 was comparable to that of heat-killed gut LPLs. **(C)** Human gut LPLs were treated with carbamazepine (20μM, 100μM, 500μM), everolimus (2nM, 5nM, 10nM), rapamycin (20nM, 100nM, 500nM) for 48 hours, or medium control, or heat-killed by exposing to 56°C for 10 minutes. Cell viability was subsequently determined using the CellTitre-Glo® 3D Cell Viability Assay (Promega cat. #G9681) according to the manufacturer's instructions. Viability of cells treated with medium control was set at 100%. Selected concentrations for experiments using intestinal LPLs were 100nM carbamazepine, 5nM everolimus, and 100nM rapamycin. **(B-C)** Data are representative of n=2 donors measured in duplicate. Open circles represent the mean of duplicates from independent donors. **(D)** Singlet, live cells were first gated on CD3 expression. CD11c+ DCs, identified as CD3-CD11c+ cells, were negligible in number. CD4+ T cells were identified as CD3+CD11c- cells, and represented the majority of T cells. **(E)** For our prophylactic treatment model using intestinal LPLs, LPLs were plated in a 96-well plate, and pre-incubated with selected autophagy drugs (day 0). 15 hours later (day 1), LPLs were infected with HIV-1 NL4.3BaL. After 72 hours of culture (day 3), LPLs were extensively washed to remove unbound virus, and co-cultured with the HIV-1 permissive U87.CD4.CCR5 cell line for an additional 72 hours (day 6). **(F)** For our therapeutic treatment model using intestinal LPLs, cells were first infected with HIV-1 NL4.3-BaL (day 0), 15 hours prior to treatment with selected autophagy drugs (day 1). After 72 hours of culture (day 3), LPLs were extensively washed to remove unbound virus, and co-cultured with the HIV-1 permissive U87.CD4.CCR5 cell line for an additional 72 hours (to day 6). **(E-F)** HIV-1 transmission by LPLs was assessed in LPL-U87.CD4.CCR5 co-culture, determined by intracellular p24 staining by flow cytometer.

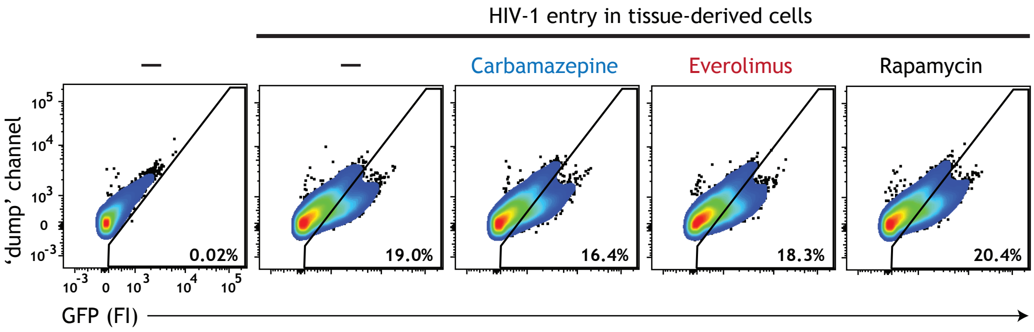

Supplementary Information Figure S8. Autophagy drugs carbamazepine, everolimus, and rapamycin do not impact HIV-1 entry in tissue-derived cells. Human intestinal lamina propria lymphocytes (LPLs) were isolated as previously described [39]. LPLs were treated with carbamazepine (100μM), everolimus (5nM), or rapamycin (100nM), or left untreated, and subsequently infected with fluorescently-tagged HIV-1 Gag-iGFP virus [NIH AIDS reagents, catalog no. 12457] for 48 hours (MOI = 0.7). HIV-1 entry was determined by quantifying GFP signal in infected tissue-derived cells by flow cytometry. Representative flow cytometry plots of n=2 donors demonstrate no effect of drug treatment on virus entry.
